# Using Spectral Blurring to Assess Effects of Channel Interaction on Speech-in-Noise Perception with Cochlear Implants

**DOI:** 10.1007/s10162-020-00758-z

**Published:** 2020-06-09

**Authors:** Tobias Goehring, Julie G. Arenberg, Robert P. Carlyon

**Affiliations:** 1 Cambridge Hearing Group, Medical Research Council Cognition and Brain Sciences Unit, University of Cambridge, 15 Chaucer Road, Cambridge, CB2 7EF UK; 2 Massachusetts Eye and Ear, Harvard Medical School, 243 Charles St, Boston, MA 02114 USA

**Keywords:** cochlear implants, speech perception, channel interaction, site selection

## Abstract

Cochlear implant (CI) listeners struggle to understand speech in background noise. Interactions between electrode channels due to current spread increase the masking of speech by noise and lead to difficulties with speech perception. Strategies that reduce channel interaction therefore have the potential to improve speech-in-noise perception by CI listeners, but previous results have been mixed. We investigated the effects of channel interaction on speech-in-noise perception and its association with spectro-temporal acuity in a listening study with 12 experienced CI users. Instead of attempting to reduce channel interaction, we introduced spectral blurring to simulate some of the effects of channel interaction by adjusting the overlap between electrode channels at the input level of the analysis filters or at the output by using several simultaneously stimulated electrodes per channel. We measured speech reception thresholds in noise as a function of the amount of blurring applied to either all 15 electrode channels or to 5 evenly spaced channels. Performance remained roughly constant as the amount of blurring applied to all channels increased up to some knee point, above which it deteriorated. This knee point differed across listeners in a way that correlated with performance on a non-speech spectro-temporal task, and is proposed here as an individual measure of channel interaction. Surprisingly, even extreme amounts of blurring applied to 5 channels did not affect performance. The effects on speech perception in noise were similar for blurring at the input and at the output of the CI. The results are in line with the assumption that experienced CI users can make use of a limited number of effective channels of information and tolerate some deviations from their everyday settings when identifying speech in the presence of a masker. Furthermore, these findings may explain the mixed results by strategies that optimized or deactivated a small number of electrodes evenly distributed along the array by showing that blurring or deactivating one-third of the electrodes did not harm speech-in-noise performance.

## Introduction

Cochlear implants (CIs) provide a sense of sound for people with severe-to-profound hearing loss by electrically stimulating the auditory nerve with an array of electrodes. While CI users achieve good speech understanding in quiet acoustic conditions, most of them struggle to understand speech in noise (e.g., Friesen et al. 2001; Cullington and Zeng 2008). Efforts to improve speech-in-noise perception by applying speech enhancement or noise reduction techniques within the CI speech processor have provided some benefits but are still limited to conditions with spatially separated signals, stationary noise, or a priori knowledge about the noise characteristics (Dawson et al. 2011; Hersbach et al. 2012; Goehring et al. 2017, 2019a). Even with state-of-the-art algorithms, CI users struggle in moderate levels of background noise and remain strongly affected by the detrimental effects of current spread and the resulting interactions between electrode channels (e.g., Carlyon et al. 2007; Oxenham and Kreft 2014). The limitations in speech-in-noise perception by CI users are likely due to interactions between the different electrodes, each of which is used to convey information about a specific frequency region of the incoming sound (Henry et al. 2000; Nelson et al. 2011). It has been suggested that these channel interaction effects account for the lack of improvement in CI speech perception as the number of channels increases above about 4–10 (Dorman and Loizou 1997; Hanekom and Shannon 1998; Friesen et al. 2001; Fu and Nogaki 2005). However, recent studies have provided some evidence that for state-of-the-art CIs with more electrode sites than used in earlier studies, benefits still increased beyond about 8–12 channels for some device types and electrode configurations (Croghan et al. 2017; Schvartz-Leyzac et al. 2017; Berg et al. 2019a), while this was not the case for some other types (Berg et al. 2019b). These findings show that channel interaction remains an important limitation for CI hearing and that further research is required to better understand and potentially ameliorate its effects on speech-in-noise perception.

Previous studies have used normal-hearing (NH) listeners and noise- or tone-based envelope vocoder schemes to simulate the effects of current spread by varying the spectral overlap between channels and/or by changing the total number of channels (Qin and Oxenham 2003; Fu and Nogaki 2005; Litvak et al. 2007; Bingabr et al. 2008; Crew and Galvin 2012; Oxenham and Kreft 2014; Mesnildrey and Macherey 2015; Grange et al. 2017; Jahn et al. 2019). In those studies, spectral overlap between channels with slopes of about − 12 dB/octave and about 4–8 channels led to similar results between speech-in-noise performances by NH listeners using vocoders and CI users. Those findings supported the rationale that substantial channel interaction effects due to current spread can account for the difficulties in speech-in-noise perception with CIs. This is in line with studies performed with CI users that reported strong channel interaction effects (Hanekom and Shannon 1998) and that found speech-in-noise perception mostly unaffected by changes to the analysis filter bank (Loizou et al. 2000; Fu and Shannon 2002). For example, Fu and Shannon (2002) introduced spectral smearing between the analysis frequency bands of a four-channel CI processor by varying the slopes of the analysis filters and found that speech performance was unaffected for slopes of − 12 dB/octave or steeper. This is consistent with the slope found for spatial tuning curves in CI users (Nelson et al. 2011) and the slope values used in NH vocoder studies to simulate performance of CI users (Oxenham and Kreft 2014). However, the use of a very small number of electrode channels in those older studies limits the applicability of the findings to state-of-the-art cochlear implant devices that use between 12 and 24 electrode channels. It remains unclear if speech-in-noise perception is affected differently by channel interaction for the case with a large number of closely spaced electrodes over the case with a small number of spatially more separated electrodes used in earlier studies.

Numerous attempts have been made to reduce channel interaction effects at the level of the electrode channels used for stimulation to improve speech-in-noise perception with CIs. These studies can be split into two main groups: current focusing and site-selection strategies.

Researchers have attempted to alleviate channel interaction effects by using current focusing to more precisely excite the targeted neural regions (Mens and Berenstein 2005; Berenstein et al. 2008; Srinivasan et al. 2013; Bierer and Litvak 2016; Langner et al. 2017; De Jong et al. 2019). Current-focussing methods simultaneously stimulate a number of adjacent electrode channels using a pattern of positive and negative current to control and narrow the shape of the neural excitation profile (van den Honert and Kelsall 2007; Bierer 2007; Bonham and Litvak 2008). However, results for speech perception have been at best mixed, with one study reporting improvements at group level (Srinivasan et al. 2013) while the majority did not find benefits (Mens and Berenstein 2005; Berenstein et al. 2008; Bierer and Litvak 2016; Langner et al. 2017; DeVries and Arenberg 2018; De Jong et al. 2019). It is possible that the use of acute testing without extended periods of acclimatization may have led to an advantage for the control condition and confounded the results somewhat. Consistent with this idea, Srinivasan et al. (2013) reported superior performance for a focused strategy compared to an experimental monopolar strategy, both of which differed from that used clinically by the participants. DeVries and Arenberg (2018) also compared two strategies that both differed from the clinical strategy, and found better performance when focussing electrode channels furthest away from the auditory neurons than when focussing other electrodes. Overall, there is little evidence that current focusing translates into benefits in speech perception for CI users.

Another attempt to reduce channel interaction has been to deactivate a number of electrode channels. These site-selection strategies deactivated a subset of electrodes with the goal of reducing channel interaction effects that may arise from the interference from “bad” channels with wide excitation patterns (Zwolan et al. 1997; Noble et al. 2013, 2014; Saleh et al. 2013; Bierer and Litvak 2016; Garadat et al. 2013; Vickers et al. 2016; Zhou 2017; Goehring et al. 2019b). Typically, these studies deactivated about one-third of the available electrode channels to keep a minimum number of active electrodes required for speech-in-noise perception. The deactivated electrode channels were chosen based on channel-wise measures related to electrode-nerve-distance, presumed local neural health of the auditory nerve, single-channel modulation detection or spread of excitation. Most studies additionally implement a rule that prevents multiple adjacent channels from being deactivated, with the result that the deactivated channels are spread fairly evenly along the array. Electrode channels that are far from the auditory nerve or that lie close to a neural dead region are likely to have a wider excitation pattern and therefore interfere more with neighbouring channels. The results were mixed with some studies reporting improvements in speech perception over a control condition (Garadat et al. 2013; Saleh et al. 2013; Noble et al. 2013, 2014) while others did not find improvements (Vickers et al. 2016; Bierer and Litvak 2016; Goehring et al. 2019b). The use of different measures and criteria between studies to select electrode channels for deactivation could have led to the mixed results. Furthermore, it is likely that different measures of electrode-nerve distance and presumed neural health along the cochlea interact and influence the spread of excitation in a complex manner so that the optimal selection of electrodes for improving speech-in-noise performance is not obvious (Brochier et al. 2020). Generally, all of the site-selection studies each used only a single measure to guide the selection of deactivated channels without taking into account all the aspects of electrode-nerve distance, local neural health and their interactions. It remains an open question how these different aspects of channel interaction contribute to speech-in-noise perception and whether more complex assessments of the electrode-nerve interface can be used to improve performance using site-selection strategies. In general, there is a limited understanding of how channel interactions affect speech performance with state-of-the-art cochlear implants that utilize a large number of closely spaced electrodes and of which measures or criteria are best suited to identify and deactivate channels for optimizing performance. In particular, the extent to which individual electrode channels contribute to the complex relationship between channel interactions and speech perception remains unclear.

All channel deselection approaches make at least two, often implicit, assumptions. The first is that there are some “bad” electrode channels that, in the standard clinical map, convey inaccurate or distorted information about the signal. The second is that this interferes in some way with the processing of information conveyed by other, “good” electrode channels, which is why deactivating the bad electrodes should help. The nature of this interference is not always stated but could include peripheral masking due to current spread and/or charge interactions, or perhaps more central processes being unable to “ignore” the spectral distortions due to bad channels. Here we test these two assumptions for one particular form of distortion—spectral blurring—and for the case where we simulate blurring one-third of the electrodes, evenly spaced along the array. Blurring the representation of the frequency spectrum has been proposed in several studies as one way in which bad channels might degrade speech perception, which in turn may be improved by their deselection or reprogramming (e.g. Zwolan et al. 1997; Noble et al. 2013, 2014). However, as mentioned above, it still remains elusive how to identify bad electrode channels in CIs which led us to artificially introduce bad electrode channels in a controlled manner. We chose spectral blurring to manipulate the bad electrode channels as it has been proposed as one mechanism in which speech perception with CIs may be degraded and because it was possible to simulate its effects, as described below. The intention was to measure the detrimental effect that a set of bad channels can have on CI users’ speech perception and to determine whether deactivation of the, in our case explicitly known, bad channels can reverse this drop in performance. We chose to simulate blurring on five evenly spaced electrodes along the array because most channel deselection and reprogramming strategies imposed some constraints so as to avoid the deselection of groups of adjacent channels (e.g. Garadat et al. 2012, 2013; Zhou 2016, 2017; Goehring et al. 2019b).

Two types of spectral blurring were administered. Experiment 1 simulated blurring at the input to the CI by changing the spectral overlap between analysis filters. Experiment 2 simulated blurring at the output of the CI speech processor, by conveying the envelope of individual channels using groups of adjacent electrodes, stimulated simultaneously and in phase. As noted above, in one set of conditions of each experiment, this blurring was applied to one-third of channels evenly spaced along the array. In another set of conditions, the blurring was applied to all electrodes. This latter set of conditions served three purposes. The first was to check that the amounts of spectral blurring used were large enough to affect performance even when applied to all electrodes; if not one could hardly expect them to do so when applied only to a subset of electrodes. Second, we wished to determine, at the group level, how much blurring was needed to degrade performance. The third purpose was to use the function relating performance to the amount of blurring to obtain an estimate of spectro-temporal resolution on a listener-by-listener basis. The rationale was based on the “equivalent noise method” which has a long history in vision research and has also been used in auditory research (Lu and Dosher 1998; Pelli and Farell 1999; Ihlefeld and Sanes 2015). Specifically, we assume that there is an internal amount of blurring and that performance should be degraded only when any blurring applied to the stimulus exceeds this amount. This assumption can be viewed as a consequence of the fact that reducing blurring in the stimulus is unlikely to improve performance if it is followed by substantial internal blurring, such as could be caused by current spread or by more central, neural, processes. We therefore measured performance as a function of the amount of blurring, and used the blurring amount at which performance started to deteriorate as a measure of the spectro-temporal resolution for each subject. We then correlated this estimate, across listeners, with performance on a non-speech spectro-temporal task (Archer-Boyd et al. 2018, 2020).

The main hypothesis was that increasing amounts of spectral blurring degrade speech-in-noise performance at group level. The second hypothesis was that there is a negative relationship between the effects of spectral blurring on speech-in-noise performance and spectro-temporal resolution at an individual level, such that CI listeners with a poorer spectro-temporal acuity will be affected by larger amounts of blurring and vice versa. The third hypothesis was that deactivating a set of “bad” electrodes, in this case the 5 electrode sites that were blurred, would recover speech performance to the level of the control condition without blurring. To answer these questions, we performed two listening experiments with a group of experienced CI users and used spectral blurring to manipulate the channel interaction between electrode channels either on all or one-third of the electrode sites.

## Methods

### Subjects

Twelve post- and peri-lingually deafened, native speakers of British English took part. Half of them were female and their mean age was 67 years, with a range from 49 to 76 years. Subjects were unilaterally implanted users of an Advanced Bionics (“AB”; Valencia, CA, USA) HiRes 90K™ cochlear implant and had at least 3 years of experience with their device with a mean duration of implant use of 5.8 years. Only the implanted ear of each subject was used for the presentation of stimuli. If a subject was wearing a hearing aid in the other ear, then it was taken off during the experiment. Prior to the experiment, the most recent clinical MAP was obtained for each subject. Details about the demographic information and devices used by the subjects are given in Table 1. The study was approved by the National Research Ethics committee for the East of England. Subjects gave their informed consent and were paid for taking part and reimbursed for travel expenses. All twelve subjects took part in experiment 1 (“Experiment 1: Blurring at the Input Level”) out of which eight took also part in experiment 2 (“Experiment 2: Blurring at the Output Level”) several months later.

**Table 1:** Subject demographics and devices

Subject	Specifier	Sex	Age (years)	Duration implanted (years)	Duration of profound hearing loss (years)	CI speech processor	CI electrode array	Clinical CI strategy, pulse width (μs)	Deactivated electrodes in clinical MAP
S1	AB3	M	72	11	36	HR90K Naida	HiFocus 1J	HiRes Optima-S, 29.6	–
S2	AB1	M	74	10	41	HR90K Harmony	HiFocus 1J	HiRes Optima-S, 26	16
S3	AB6	F	70	5	65	HR90K Naida	HiFocus 1J	HiRes Optima-S, 35	16
S4	AB24	F	49	3	4	HR90K Advantage	HiFocus MS	HiRes Optima-S, 35	16
S5	AB26	F	58	4	21	HR90K Advantage	HiFocus MS	HiRes Optima-S, 22.4	–
S6	AB23	F	60	3	58	HR90K Advantage	HiFocus MS	HiRes Optima-S, 23.3	–
S7	AB25	F	66	3	34	HR90K Advantage	HiFocus MS	HiRes Optima-S, 18	16
S8	AB2	F	60	11	27	HR90K	HiFocus 1J	HiRes Optima-S, 31.4	16
S9	AB20	M	73	3	40	HR90K Naida	HiFocus MS	HiRes Optima-S, 29.6	–
S10	AB19	M	75	3	Unknown	HR90K Naida	HiFocus MS	HiRes F120, 18	–
S11	AB05	M	76	9	27	HR90K Harmony	HiFocus 1J	HiRes Optima-S, 30.5	8
S12	AB09	M	73	5	Unknown	HR90K Naida	HiFocus MS	HiRes Optima-S, 35	–

### Speech-in-Noise Test

Speech-in-noise (SIN) performance was tested following the procedure described by MacLeod and Summerfield (1990). Sentence lists from the BKB corpus (Bench et al. 1979) spoken by a British male talker were mixed with time-reversed speech from the Harvard sentences (Rothauser 1969) spoken by a different British male talker. This background noise contained the highly modulated characteristics of competing speech, as it occurs in realistic listening environments, but reduced informational masking with the use of an unintelligible masker (Deeks and Carlyon 2004). Furthermore, the use of a competing talker as masker has been suggested to be more sensitive to differences in spectral resolution by CI users than other masker types such as stationary speech-shaped noise (Croghan and Smith 2018). An adaptive one-up/one-down procedure was used to measure the speech reception threshold (SRT) at which 50 % of the sentences were understood correctly. The initial signal-to-noise ratio (SNR) was set to 4 dB SNR, and increased by 2 dB per trial, while repeating a randomly drawn sentence from the list, until the subject repeated the three keywords correctly. The adaptive procedure adjusted the SNR with a fixed step size of 2 dB until all 15 sentences of that list had been presented. A trial was deemed correct if all three keywords of that sentence were correctly repeated by the subject and the final SRT score for that run was calculated as the average of the last ten SNRs presented.

### Spectro-temporal Test

Each subject’s spectro-temporal acuity was evaluated using the Spectro-Temporal Ripple for Investigating Processor EffectivenesS test (STRIPES, Archer-Boyd et al. 2018). The STRIPES test uses an adaptive procedure to measure the threshold at which the subject can just distinguish the target stimulus from two reference stimuli in a three-interval, two-alternative forced-choice task. Stimuli consisted of 1-s-long, concurrent exponential sine sweeps moving up or down in frequency from 250 to 8000 Hz. The subject had to select the target interval, which was either the first or last interval, and which was always an upward sweep; the other two intervals contained downward sweeps. The number of concurrent frequency sweeps (the “density”) is varied to titrate difficulty, with the task being very easy at a density close to 1, and progressively harder at higher densities. The starting frequency was roved across trials and the beginning and end of each interval was masked by short noise bursts to reduce the salience of onset and offset cues. An adaptive two-up/one-down procedure started with a sweep density of 1.1 and adjusted the density per trial with a density step size of 0.5 (for the first 4 reversals) and 0.2 (for the last eight reversals). (A non-integer density can occur, when, in this example, 2 stripes are present during 10 % of the time and 1 stripes is present 10 % of the time; see Archer-Boyd et al. (2018) for details.) The test was complete after 12 reversals and the final score of the run was calculated as the average of the last four reversals. Each subject performed three runs of the STRIPES test with the experimental MAP that was most similar to their clinical MAP and their STRIPES score was calculated as the average of those three scores. The STRIPES test takes about 5–10 min per run, leading to a total of about 30 min of testing per subject.

### Technical Setup

The experiment took place in a sound-attenuated testing room. Testing stimuli for the SIN and STRIPES tests were presented with a laboratory owned programmable Harmony CI speech processor (Advanced Bionics, US) that was worn by the subjects during the testing. Stimuli were generated in MATLAB (Mathworks, US) using a battery-powered laptop computer (Dell XPS15, Windows 10 Pro) that was connected via an external soundcard (Roland UA-55) and an audio cable to the auxiliary input of the CI speech processor. For each subject and at the beginning of each SIN testing session, the presentation level was set by adjusting the manual volume control of the soundcard to a “comfortable level” for each subject (level 6 on the loudness scale provided by Advanced Bionics) using a sentence from the Harvard lists. All SIN stimuli were calibrated to the same RMS level as the stimuli used to set the presentation level and the presentation level was kept the same for all MAPs under test. For safety reasons and to confirm equal loudness between MAPs, subjects were asked during the testing if the presentation level was comfortable to them for each of the different MAPs and this was confirmed by all subjects and for all MAPs.

## Experiment 1: Blurring at the Input Level

The first experiment investigated the effects of channel interaction at the input level of the CI (analysis filter stage) on speech perception in noise. All 12 subjects participated in this part of the experiment. Here we altered the spectral overlap of the input filters to indirectly simulate current spread effects at the later stages of the stimulation.

### Method for Blurring at the Input Level

**Fig. 1:**
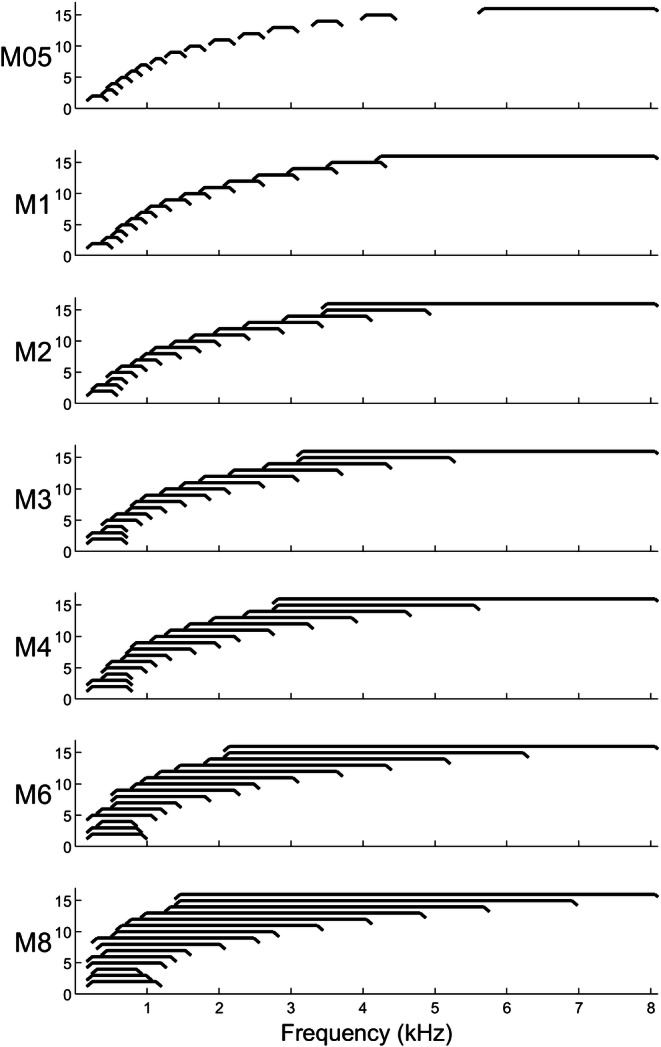
Blurring at the input level: visualization of the filter channel output bandwidths as described in Table 2 for the experimental MAPs in the ALL-15-Input condition (x axis indicates the channel number). Each filter’s bandwidth is indicated by the horizontal lines between the rising and falling slopes with edges showing the lower and upper cut-off frequencies. Note that the curve used here to show each filter bandwidth is a simplification and does not represent the spectral transfer function of the filter. A small offset is applied on the vertical axis (no unit) from apical (bottom, left) to basal (top, right) filters to allow for better visibility of the individual filter bandwidths. For M6 and M8, the mapping of frequency channel bandwidths led to non-monotonic increases in lower and upper cut-off frequencies across filter channels 2 to 9 (see also Table 2 for comparison)

For the first experiment, we varied the amount of blurring, as a means of manipulating channel interaction, by adjusting the spectral overlap between electrode channels in terms of the acoustic bandwidths of the input analysis filters. This method of blurring at the input of the CI has been used previously and led to significant decreases in performance on a spectro-temporal test with CI users (Archer-Boyd et al. 2018). The Advanced Bionics (AB) CI speech processor uses a 16-band analysis filter bank with a frequency range from 238 up to 8054 Hz. The filter bank channels are constructed by combining sets of output bins obtained from an FFT analysis stage and do not normally overlap with adjacent channels in the standard clinical MAP. Using the standard clinical MAP for each subject as starting point, we generated a set of experimental MAPs per subject by changing the lower and upper cut-off frequencies of the individual filter bank channels (using BEPS+ software from AB), thereby decreasing or increasing the spectral overlap between adjacent channels. The centre frequencies of the filter channels were kept roughly constant between all MAPs, and only the bandwidths of the “blurred” channels were multiplied by a factor of 0.5, 2, 3, 4, 6 or 8 (Table 2). This led, for example in the case of a blurring factor of 8, to filter bandwidths that were 8-fold wider than in the standard clinical MAP. We compensated for the change in filter bandwidth by applying a correction gain for each filter channel to compensate for changes in loudness. This gain was calculated as ten times the base-10 logarithm of the ratio of the blurred and original bandwidths in Hz. Six different blurring factors were used to generate 12 experimental MAPs in total. Half of those were generated by applying the spectral blurring to all 15 active electrode channels (condition “ALL-15-Input”, shown in Fig. 1) and half were generated by blurring 5-of-15 active electrode channels (condition “5-of-15-Input”) that were distributed evenly along the array with a spacing of 3 electrodes. For the 5-of-15-Input condition, we changed the filter bandwidths of only five electrodes, either electrodes 2,5,8,11,14 or electrodes 3,6,9,12,15, to the same as used in the ALL-15-Input conditions with the remaining electrodes kept similar as for M1 or the clinical MAP. The two 5-of-15-Input electrode selections were equally split between subjects. In the following, the MAP most similar to the clinical MAP is noted as “M1” and the experimental MAPs are noted as “M05, M2, M3, M4, M6, M8”, for the ALL-15-Input condition, and “M05b, M2b, M3b, M4b, M6b, M8b”, for the 5-of-15-Input condition, whereas the MAP number directly reflects the blurring factor used in that MAP.

**Table 2:** Lower and upper filter cut-off frequencies used for the experimental MAPs for blurring at the input level. Note that for M6 and M8, the mapping of frequency channel bandwidths led to non-monotonic increases in lower and upper cut-off frequencies across filter channels 2 to 9 (see also Fig. 1 for comparison)

Filter channel	M05		M1		M2		M3		M4		M6		M8	
	Low	High	Low	High	Low	High	Low	High	Low	High	Low	High	Low	High
1	238	374	238	442	238	510	238	646	238	714	238	918	238	1122
2	442	510	442	578	306	578	238	646	238	714	238	850	238	986
3	510	578	578	646	510	646	442	646	442	714	374	782	306	850
4	646	714	646	782	510	782	442	850	442	918	238	1054	238	1190
5	782	850	782	918	646	918	578	986	510	1054	374	1190	238	1326
6	918	986	918	1054	850	1122	782	1190	714	1258	578	1394	442	1530
7	1122	1190	1054	1257	986	1394	850	1462	782	1598	578	1802	374	2006
8	1326	1462	1257	1529	1122	1666	986	1802	850	1938	578	2210	306	2482
9	1598	1734	1529	1801	1394	1938	1258	2074	1122	2210	850	2482	578	2754
10	1938	2142	1801	2141	1666	2346	1530	2550	1326	2686	986	3026	646	3366
11	2346	2550	2141	2549	2006	2822	1802	3026	1598	3230	1190	3638	782	4046
12	2754	3026	2549	3025	2414	3366	2210	3638	1938	3842	1462	4318	986	4794
13	3366	3638	3025	3568	2958	4046	2686	4318	2414	4590	1870	5134	1326	5678
14	4046	4386	3568	4248	3502	4862	3162	5202	2822	5542	2142	6222	1462	6902
15	5678	8058	4248	8054	3502	8058	3162	8058	2822	8058	2142	8058	1462	8058

It should be noted that electrode 16 was deactivated for all subjects in this experiment to make the experimental MAPs as similar as possible across subjects. We also never used electrodes that were deactivated in the subject’s clinical MAP, which affected only subject S11 with electrode 8. For S11, this led to a gap in the stimulation pattern of all test conditions due to electrode 8 being disabled. We used electrodes 3,6,9,12,15 for the 5-of-15-Input condition with S11 to keep the same number of five manipulated channels as for the other subjects. Furthermore, the processing strategy was changed to HiRes-S for all subjects (similar to continuous interleaved sampling, CIS, without current steering or noise reduction functions active as compared to their clinical MAP). The same pulse rate and pulse width was used between the different experimental MAPs but differed between subjects depending on their clinical settings.

### Experimental Procedure

Experiment 1 was split into two 3-h sessions per subject that were performed on two different days. In the first session, subjects completed the SIN test first with five MAPs from the ALL-15-Input condition (M05, M1, M2, M3, M4) in random order and second with five MAPs from the 5-of-15-Input condition (M05b, M1b, M2b, M3b, M4b) in random order. For each MAP and before the SIN test, subjects were presented with one randomly chosen list (10 sentences) from the Harvard sentences and were able to read along to acclimatize to that MAP. The SIN test was then performed twice per MAP and the average of the two runs was taken as the final SRT for that MAP. In the second session, subjects were tested with three MAPs from the ALL-15-Input conditions (M4, M6, M8) in random order and then with three MAPs from the 5-of-15-Input condition (M4b, M6b, M8b) in random order. The same procedure as in the first session was followed. The scores from the two sessions were used together for the analysis, with the score for M4 and M4b (which were tested in both sessions) calculated as the average between the two sessions. After the SIN testing was complete, subjects performed three runs of the STRIPES test with M1 and the average was taken as their final STRIPES score.

### Results

**Fig. 2:**
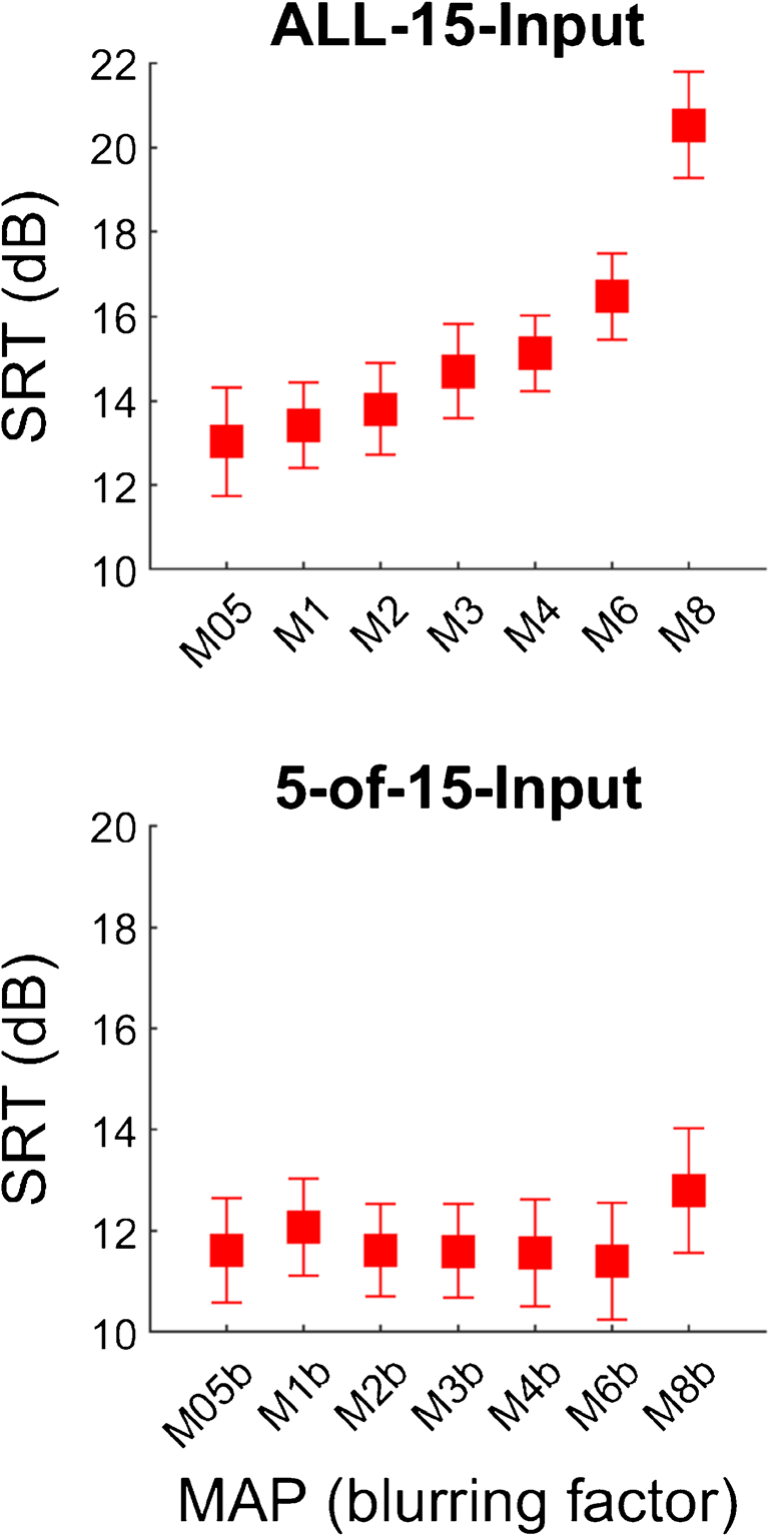
SRT group averages for blurring at the input level for using ALL-15-Input (top) and 5-of-15-Input (bottom) electrode channels. Error bars depict standard errors

Group average scores for the SIN test with blurring at the input level are shown in Fig. 2 for the ALL-15-Input and the 5-of-15-Input conditions. The effect of blurring was evaluated using one-way repeated-measures ANOVAs with the factor MAP for each condition. For the ALL-15-Input condition, there was a significant main effect of MAP [*F*(6,66) = 19.68, *p* < 0.001]. Pairwise comparisons using Tukey’s honest significance test revealed significant differences for the comparisons M05 vs M3 (Diff = 1.7 dB, *t* = −3.19, *p* < 0.05), M05 vs M4 (Diff = 2.1 dB, *t* = −3.81, *p* < 0.01), M05 vs M6 (Diff = 3.4 dB, *t* = −3.83, *p* < 0.01) and M05 vs M8 (Diff = 7.5 dB, *t* = −7.1, *p* < 0.01); for M1 vs M4 (Diff = 1.7 dB, *t* = −3.42, *p* < 0.05) and M1 vs M8 (Diff = 7.1 dB, *t* = −6.63, *p* < 0.01); for M2 vs M6 (Diff = 2.7 dB, *t* = −3.17, *p* < 0.05) and M2 vs M8 (Diff = 6.7 dB, *t* = −6.73, *p* < 0.01); for M3 vs M8 (Diff = 5.8 dB, *t* = −5.67, *p* < 0.01); and for M6 vs M8 (Diff = 4.1 dB, *t* = −4.4, *p* < 0.01). These differences in SRTs were close to or substantially larger than 2 dB which is considered a clinically relevant difference for the speech-in-noise test used here. For the 5-of-15-Input condition, there was no significant main effect of MAP [*F*(6,66) = 0.72, *p* = 0.634].

**Fig. 3:**
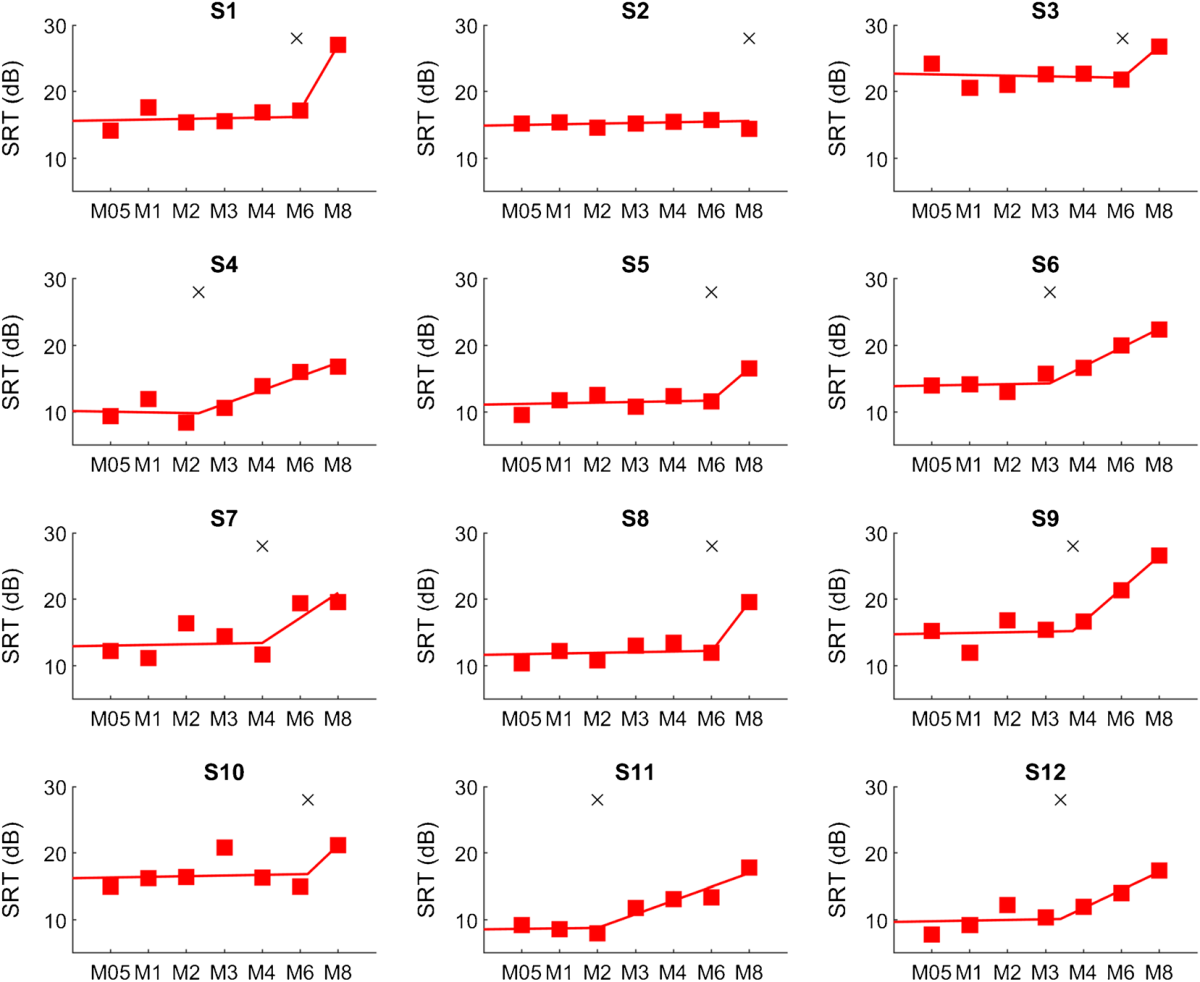
Subject-wise SRTs for blurring at the input level with the ALL-15-Input condition. A two-segment linear regression was fitted to the SRTs of each subject to derive the knee points (indicated by a cross)

The individual SIN scores for the twelve subjects and all MAPs are shown in Fig. 3 for the ALL-15-Input condition. A segmented linear regression with two segments was fitted using the “fit” function provided by MATLAB (Mathworks, US). The first segment was restricted to very small slope values in the range [− 0.1, 0.1] (change in SRT per blurring factor) and the second segment was restricted to positive slopes in the range [0, 20]. There were no further restrictions applied and the same settings were used for all subjects. The knee points of the two segments were considered the threshold at which spectral blurring affected speech-in-noise perception at the subject level. Knee points varied markedly between subjects and ranged from a spectral blurring factor of 2 for S11 up to 8 for S2 (the maximum value possible), with the other subjects in between these extremes. Interestingly, subject S2 showed no detrimental effect of spectral blurring for any MAP, but all other subjects had knee points smaller than 8. There was a positive relationship between spectral blurring knee points and SIN performance with M1 across subjects (*r* = 0.63, *df* = 10, *p* = 0.029), meaning that subjects with better SIN performance (lower score) with a MAP that was most similar to their everyday setting were more affected by spectral blurring (lower knee point) than subjects with worse SIN performance. This correlation was of similar size and significant even when the SRT with M1 was excluded from the calculation of the blurring knee points. Table 3 shows the performance scores with M1 for the SIN test and the spectral blurring knee points for all subjects. We also calculated the knee points for the 5-of-15-Input condition but only subject S6 had a knee point lower than the maximum which was similar to the one for the ALL-15-Input condition (M3). We conclude that, overall, the blurring knee points confirmed the absence of an effect of blurring on SIN performance with the 5-of-15-Input condition.

**Table 3:** Subject-wise blurring knee points and scores with M1 for SIN and STRIPES tests

Subject	Spectral blurring knee point	SIN with M1 (SRT dB)	STRIPES with M1 (density)
S1	5.9	17.6	4.3
S2	8.0	15.4	3.7
S3	6.0	20.6	3.7
S4	2.3	12.0	7.1
S5	6.0	11.8	7.0
S6	3.1	14.2	7.6
S7	4.0	11.2	4.1
S8	6.0	12.2	3.4
S9	3.7	12.0	3.8
S10	6.2	16.2	4.6
S11	2.0	8.6	5.5
S12	3.4	9.2	4.9

**Fig. 4:**
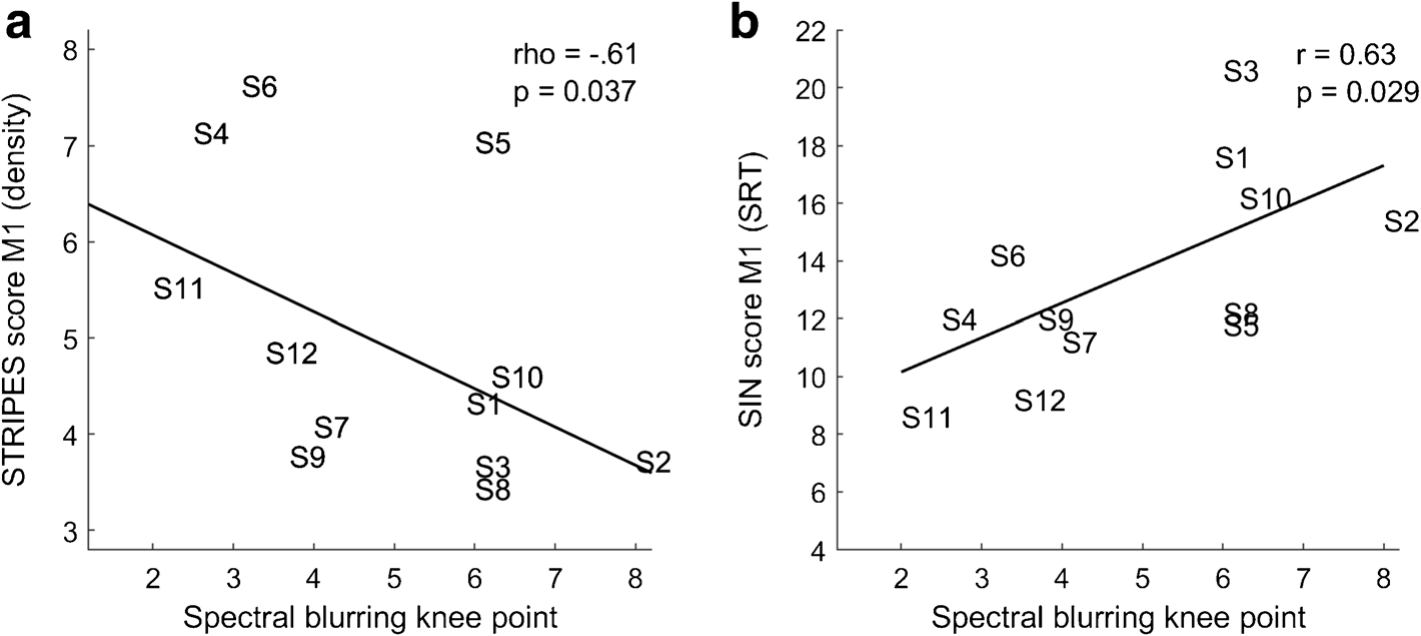
Scatter plots for the across-subject association between blurring knee points with STRIPES (**a**) and SIN scores (**b**) with M1

The results from the spectro-temporal test STRIPES are also shown in Table 3 together with the SIN M1 scores and the spectral blurring knee points. STRIPES scores varied across subjects over a range of densities from 3.4 up to 7.6 with a mean score of 5. There was a significant negative relationship between spectral blurring knee points and STRIPES scores across subjects in the predicted direction (Spearman’s rho = −0.61, *df* = 10, *p* = 0.037; a non-parametric test is used because STRIPES scores were not normally distributed according to the Shapiro-Wilk test with *W* = 0.85, *p* = 0.036). Figure 4 shows this negative association and its linear regression as well as the association between blurring knee points and SIN performance with M1.

### Discussion

We evaluated the effect of spectral blurring at the input of the CI on SIN performance in twelve CI users by adjusting the spectral overlap between electrode channels. In line with our main hypothesis, this led to an increase in SRTs with spectral blurring for the case when all electrode channels were blurred. This effect was strongest for the most extreme blurring condition M8 and statistical tests confirmed significant differences for comparisons between the conditions using no or little blurring and the conditions using higher blurring factors.

Surprisingly, there was no effect of spectral blurring on SIN performance for the case when one-third of the electrodes were blurred, even for the most extreme case with M8b. This was unexpected in light of previous research on site-specific optimization strategies in CI users, which were based on the rationale that individual electrode channels can be adjusted to alter SIN performance and, if done correctly, should lead to improvements. Here we show that even extreme blurring, when applied to five evenly spaced channels, does not impair performance on a masked speech test. Hence, at least for the particular manipulations and speech test used here, the second of the implicit assumptions described in the Introduction is not supported; performance is not reduced with five “bad” channels, even when those channels are spectrally blurred by a factor of eight. The implications and limitations of this finding and of the results of experiment 2 are discussed further in “General Discussion”.

Across-subject performance differed markedly in the ALL-15-Input condition with some subjects being more affected by spectral blurring than others. The knee points, as a measure of how much blurring was required to degrade SIN performance in a given subject, correlated with the SIN performance across subjects when using the MAP most similar to their clinical MAP. This association may have been due to an increased interference of the competing talker noise at lower SNRs than at higher SNRs, so that subjects who tolerated higher levels of noise would have been affected more by an increased overlap between filter channels than subjects who tolerated lower levels of noise. In general, this association is in line with the assumption that channel interaction affects SIN performance on an individual basis. A further advantage of measuring the knee point is that it allows one to distinguish between the effects of spectral resolution and of more central cognitive factors, both of which can affect speech perception and which are hard to disentangle when measuring performance with a single amount of (or no) blurring. Specifically, we assume that the knee point measures the limits of the listener’s internal spectral resolution, including that produced by current spread, whereas the overall SIN performance (e.g. the SRT for condition M1) reflects both spectral resolution and more central, cognitive factors.

The STRIPES test was performed to measure spectro-temporal acuity for the twelve CI subjects to explore the second hypothesis under test: subjects with high acuity, as indicated by their STRIPES scores, would be affected more by spectral blurring than subjects with low acuity. A significant correlation was indeed found. This suggested that the STRIPES test may effectively measure spectral resolution in a way that is relevant for the perception of speech. Performance on the STRIPES score did not correlate significantly with SIN performance with the M1 map (Spearman’s rho = − 0.38, *df* = 10, *p* = 0.22), but this could be due to a lack of statistical power (Anderson et al. 2011).

We note that spectral blurring at the input level, as imposed in experiment 1, simulated only some effects of the channel interactions that may arise from channels that produce broad current spread. Specifically, in the case of a subset of electrodes being affected, we simulated to some extent the loss of information conveyed by those channels, such as might occur due to neural degeneration in the auditory nerve and more centrally. However, we did not simulate the increased neural or charge interactions that occur between an electrode that produces a broad current spread and the neighbouring channels. Nevertheless, the results do show that severely degrading the information conveyed by one-third of all available electrodes has no effect on performance, and this finding should be taken into account when designing site-selection strategies based on a small subset of distributed electrodes along the array.

## Experiment 2: Blurring at the Output Level

The second experiment investigated the effects of channel interaction at the output level (electrode stage) of the CI on speech perception in noise. Eight of the subjects participated in this part of the experiment. Here, we used a more realistic simulation of current spread to alter channel interaction at the electrode level. By using several simultaneously stimulated electrodes for each channel, we directly simulated larger current spread between channels with the aim of increasing the spread of excitation and therefore the channel interaction at the level of the auditory nerve.

### Method for Blurring at the Output Level

**Fig. 5:**
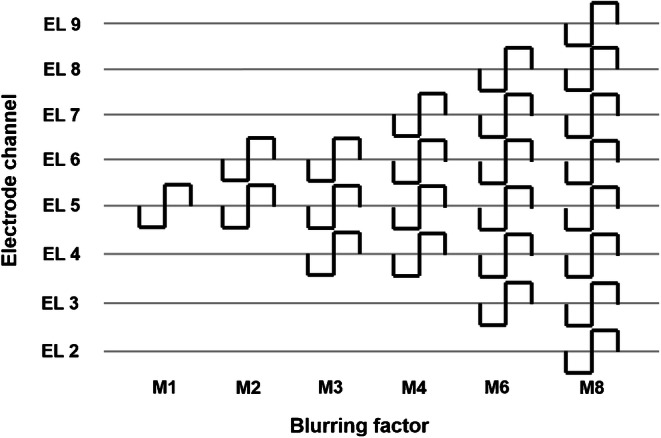
Blurring at the output level: visualization of the simultaneously stimulated electrode channels for channel 5 for the experimental MAPs. The number of simultaneous electrodes per channel increases from 1 (M1, left) to 8 (M8, right). Note that for M6 the most basal channel used only a single electrode and that M8 could only be tested for the 5-of-15-Output condition due to device limitations

For the second experiment, we varied the amount of blurring, as a means of altering channel interaction, by adjusting the number of electrodes used for the stimulation of each channel. Advanced Bionics CIs use an electrode array with up to 16 active electrode contacts for the electrical stimulation of the auditory nerve. In the standard mode as used in the clinical MAP of the subjects, either one or two electrodes are used to convey the information extracted from each of the 16 filter bank channels. A single electrode is used per filter bank channel in the CIS-like coding strategy used for the experimental MAPs (HiRes™) whereas two adjacent electrode channels, which are weighted and stimulated simultaneously to generate a virtual channel located between the two electrode channels, are used for the coding strategies that use current steering (HiRes™ Optima, HiRes™ Fidelity 120). For experiment 2, we increased the number of simultaneously stimulated electrode contacts per filter bank channel by the same factors as used for experiment 1 (with 1, 2, 3, 4, 6 and 8 simultaneous electrodes stimulated per channel for the blurring factors of 1, 2, 3, 4, 6 and 8, respectively). The same notation as in experiment 1 was used here, in which the number of the MAP indicates the blurring factor used but the conditions in experiment 2 are called “ALL-15-Output” and “5-of-15-Output” due to blurring applied at the output level of the CI. It should be noted that condition M05 was not possible here. Furthermore, while it was possible to generate those six MAPs for the 5-of-15 condition (M1b, M2b, M3b, M4b, M6b, M8b), technical reasons meant that it was not possible to generate condition M8. Therefore, only five MAPs were generated for the ALL-15-Output condition (M1, M2, M3, M4, M6). In this experiment, simultaneously stimulated electrodes were chosen so that either the middle electrode coincided with the respective filter channel (e.g. for M3 and filter channel 5, electrodes 4, 5 and 6 were used simultaneously), or they were shifted towards the basal end (e.g. for M6 and filter channel 5, electrodes 3, 4, 5, 6, 7 and 8 were used simultaneously). For all filter channels along the array, including the most basal and most apical ones, the same number of simultaneous electrodes were used (with one exception: the most basal channel in M6 used only a single electrode due to device limitations). This resulted in shifted electrode selections towards the base for the most apical electrodes (e.g. for M3 and filter channel 1, electrodes 1, 2 and 3 were used simultaneously) and shifts towards the apex for the most basal electrodes (e.g. for M3 and filter channel 15, electrodes 13, 14 and 15 were used simultaneously). The electrode selections for the MAPs used in the ALL condition are shown in Fig. 5. All other parameters of the experimental MAPs were kept the same as for M1 in experiment 1.

In addition to the experimental MAPs with blurring, we generated an experimental MAP (“Moff”) that deactivated the five electrodes that were blurred in the 5-of-15-Output MAPs. The remaining ten (or 9) electrodes that were active in Moff therefore used wider input filter bandwidths because the overall bandwidth was unchanged. Deactivating five electrodes also introduced some frequency-to-place stimulation shifts. Moff was used to compare the performance with a 10-channel MAP to the 15-channel MAPs under test, and to determine whether deactivation of the blurred channels would improve performance compared to the blurred MAPs. This condition was motivated by site-selection studies that deactivated a subset of “bad” electrodes to improve speech perception in CI users.

### Experimental Procedure

Experiment 2 was split into three 3-h sessions per subject that were performed on three different days. In the first session, subjects completed a loudness rating procedure in which the T- and M-levels were adjusted for the five electrodes that were blurred in the experimental MAPs in the 5-of-15-Output condition. After the five electrodes were adjusted individually, all active electrode channels were sequentially stimulated at M-level and re-adjusted to give similar loudness, if necessary. The SIN test was then performed with six MAPs from the 5-of-15 condition (M1b, M2b, M3b, M4b, M6b, M8b) and Moff (that had the five electrodes deactivated that were blurred in the other conditions) in random order following the same testing procedure as in experiment 1. In the second session, T- and M-levels were adjusted for all active electrodes in the experimental MAPs of the ALL-15-Output condition (M1, M2, M3, M4, M6). Again, after the individual adjustment of electrode T- and M-levels was completed, subjects listened to all active electrode channels sequentially and M-levels were re-adjusted if a channel was clearly different to the other ones. In the third session, the SIN test was then performed with five MAPs from the ALL-15-Output condition (M1, M2, M3, M4, M6) in random order and following the same procedure as before.

### Results

**Fig. 6:**
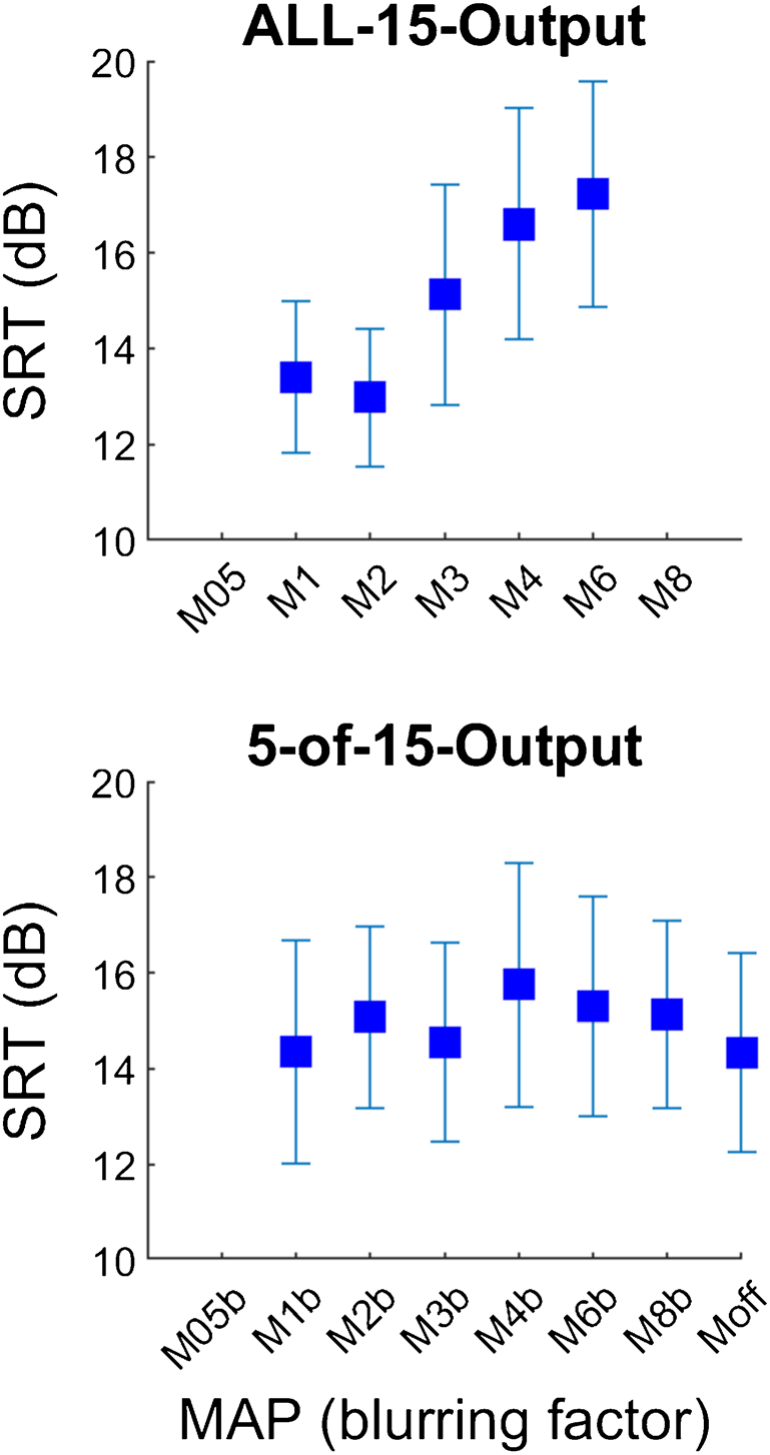
SRT group averages for blurring at the output level for using ALL-15-Output (top) and 5-of-15-Output (bottom) electrode channels. Error bars depict standard errors. Moff had five electrodes deactivated and was tested in the session with the 5-of-15 conditions

Group average scores for the SIN test with blurring at the electrode level are shown in Fig. 6 for the ALL-15-Output and the 5-of-15-Output conditions. The effect of blurring was evaluated using one-way repeated-measures ANOVAs with the factor MAP for each condition. For the ALL-15-Output condition, there was a significant main effect of MAP [*F*(4,28) = 6.81, *p* < 0.001]. Pairwise comparisons using Tukey’s honest significance test revealed significant differences for the comparisons M1 vs M4 (Diff = 3.2 dB, *t* = − 2.87, *p* < 0.05), M1 vs M6 (Diff = 3.8 dB, *t* = − 3.69, *p* < 0.01), M2 vs M4 (Diff = 3.6 dB, *t* = − 2.96, *p* < 0.05) and M2 vs M6 (Diff = 4.3 dB, *t* = − 3.19, *p* < 0.05). These differences in SRTs were all substantially larger than 2 dB and therefore considered clinically relevant effects for the speech-in-noise test used here. For the 5-of-15-Output condition, including all the blurring conditions M1b to M8b and Moff, there was no significant main effect of MAP [*F*(6,42) = 1.39, *p* = 0.242].

For this experimental part, even though there was a significant effect of blurring in the ALL-15-Output condition, no subject-wise blurring knee points could be calculated because the two-segment regression produced unstable results. This was likely due to the small number of five data points being insufficient for the fitting.

**Fig. 7:**
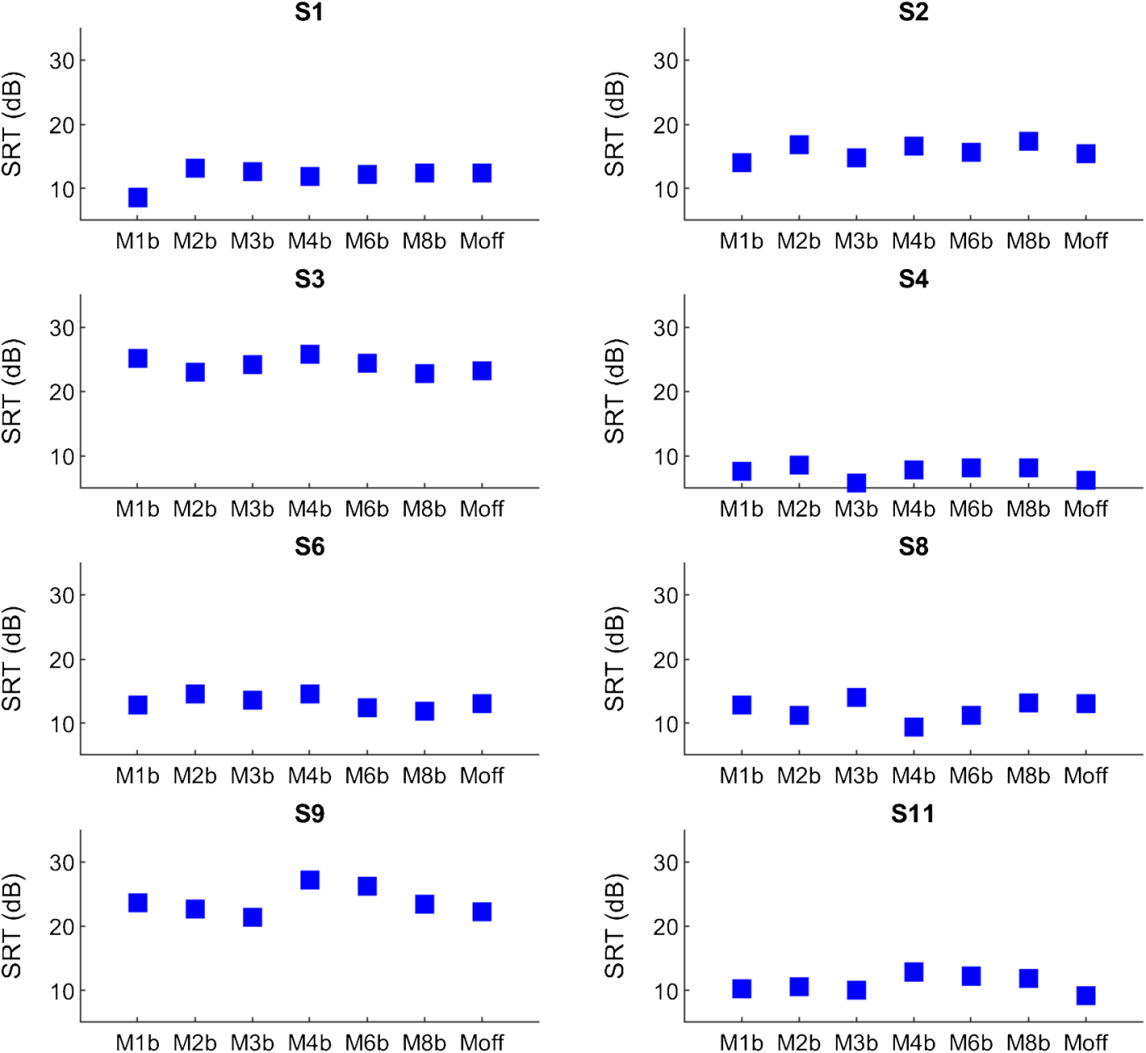
Subject-wise SRTs for blurring at the output level using 5-of-15-Output electrode channels and Moff that had those five electrodes deactivated

The statistical analysis with an RM-ANOVA for the 5-of-15-Output condition may not be sensitive enough to reveal a significant difference due to the small number of subjects (*N* = 8) and large number of comparison conditions. Figure 7 therefore shows individual results for all eight subjects for the blurred maps M1b to M8b together with Moff. It can be seen that SRTs were very similar across all conditions under test for all eight subjects. This clearly shows that there was no substantial effect on SRTs using either more blurring or the deactivation of five electrode channels in this group of subjects. Therefore, the variability between experimental conditions that can be observed in Fig. 6 is mainly due to inter-subject variability.

### Discussion

Spectral blurring at the output level, as introduced in experiment 2, simulated more directly the effects of channel interactions that arise from channels with broad current spread. We found highly significant effects of blurring on SRTs for the ALL-15-Output condition, with decreasing performance for higher amounts of blurring. This supports the rationale that blurring at the output level achieved the desired manipulation of current spread. There were no differences in performance between M1 and M2 which is in line with previous research reporting no differences in spread of excitation, pitch ranking and electrode discrimination between single- and dual-electrode stimulation modes (Hughes et al. 2013; Goehring et al. 2014), but there were highly significant differences between these and the blurring conditions using four or six electrodes. Hence blurring did not affect performance when using 2 electrodes but it did so when using 4 or more electrodes. This is in line with results reported by Langner et al. (2017) who found significant performance decreases in modulation detection thresholds and speech-in-noise intelligibility scores with a group of CI users for a “triplet” electrode configuration that used 6 simultaneously activated electrodes (3 pairs) spanning 12 electrode sites of the array (with 3 non-stimulated electrodes between the three stimulated pairs) over a standard sequential configuration with paired electrodes. Even though Langner et al.’s (2017) electrode configurations were different from blurring and they tried to compensate for channel interaction by introducing electrode gaps or flanks of opposite polarity, their findings provide further support that multi-electrode stimulation above 3 electrodes can lead to reduced spectral resolution and speech-in-noise performance. However, it cannot be excluded that other channel interaction effects such as temporal charge interactions in addition to the expected increase in spread of excitation were responsible for the decrease in performance seen in experiment 2.

In contrast to the findings with the ALL-15-Output condition, blurring of one-third of the available electrodes had no effect on performance. Hence extreme blurring either at the input or at the electrode stage did not degrade performance when applied to five evenly spaced electrodes or channels, even though it did degrade performance when applied to all electrodes. This unexpected result was confirmed both at the group level and at the subject level, with no subject showing a clear trend of decreased performance with more blurring in the 5-of-15-Output condition. Furthermore, the deactivation of the five channels that were used for the blurring led to comparable speech-in-noise scores as with the other maps. This is consistent with the finding by Friesen et al. (2001) that about 7–10 channels of information are sufficient for maximum speech-in-noise performance in CI users. In the Moff condition, subjects were able to successfully use the 10 remaining channels even with the reallocation of frequency information due to the deactivation of electrode channels. This is in line with a previous study using site selection where performance was similar between two 10-channel maps with frequency reallocation and the clinical-like map with 15 channels without reallocation (Goehring et al. 2019b). However, more recent evidence suggests that speech-in-noise performance may increase with more than 10 active electrode channels, and that this benefit may further depend on the particular configuration of the CI device, the type of electrode array and the coding strategy used (Croghan et al. 2017; Schvartz-Leyzac et al. 2017; Berg et al. 2019a, b). All of these studies used acute testing conditions and did not control for the bias effects of long-term acclimatization to the more clinical-like MAPs. In general, comparisons between studies are complicated by the use of somewhat different speech-in-noise tests that are likely to require different listening strategies composed of language, cognitive and spectro-temporal processing abilities to achieve maximum performance and are not designed to capture all aspects of speech perception.

For blurring at the electrode level, a fitting procedure was used for all active channels by adjusting the T- and M-levels for all MAPs and subjects. This ensured safe listening levels and optimized the dynamic range for each channel individually. This was necessary as the reduction in current to achieve appropriate T- and M-levels could not be predicted based on the number of active electrode sites. At group level, the levels for M2 were about half the current applied for M1, but this simple relationship did not hold for the higher blurring factors. Stimulation current ratios between M1 and the other MAPs were 1.9, 2.8, 3.5, 4.8, 5.8 for M2, M3, M4, M6, M8, respectively. The current ratios were similar between T- and M-levels with no differences larger than 0.1 apart from M8 for which the factors were 6 and 5.8 for T- and M-levels, respectively, showing that channel independence related to loudness was similar between T- and M-levels. This shows an interaction in terms of the channel-wise loudness with multiple electrodes different from a simple linear summation of current, as can be expected due to the overlap in excitation area between electrode channels. It supports the assumption that electrode channels are not independent and is consistent with the stronger effect of blurring with more simultaneously stimulated electrodes. The dynamic range values calculated as the difference between T- and M-level remained constant on a log scale across blurring conditions. This indicates that dynamic range was similar across conditions and can be excluded as potential reason for poorer SRTs with larger amounts of blurring. Interestingly, several subjects reported a change in pitch or perceived electrode channel when increasing the current from T- to M-level, especially for the strongest blurring conditions M6 and M8. This indicates that different neural regions were stimulated across the dynamic range and confirms the rationale behind blurring at the electrode level to excite broader or different neural regions than when using single-electrode stimulation. This never happened for the conditions using smaller number of electrodes M1 and M2.

## General Discussion

### Blurring Applied to All Channels

**Fig. 8:**
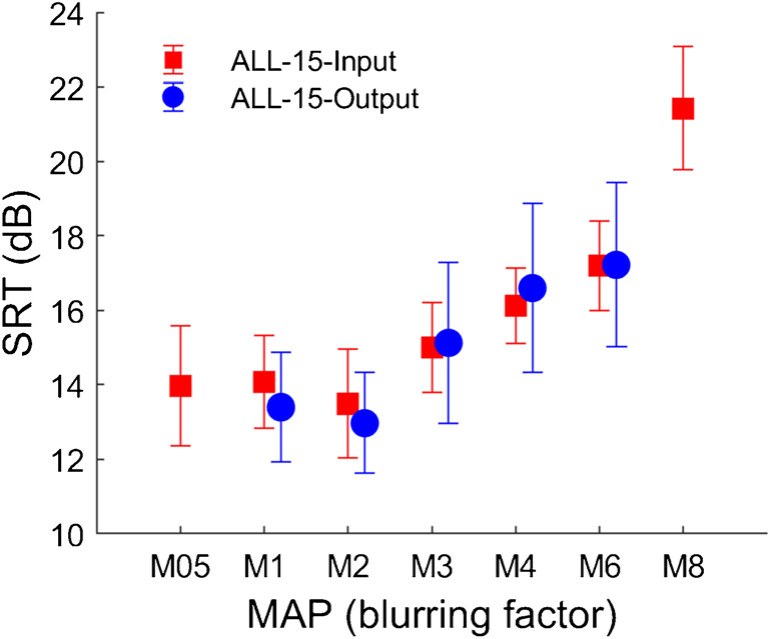
Group SRT scores in the ALL-15 condition for the eight subjects that took part in both exp. 1 (red) and exp. 2 (blue)

**Fig. 9:**
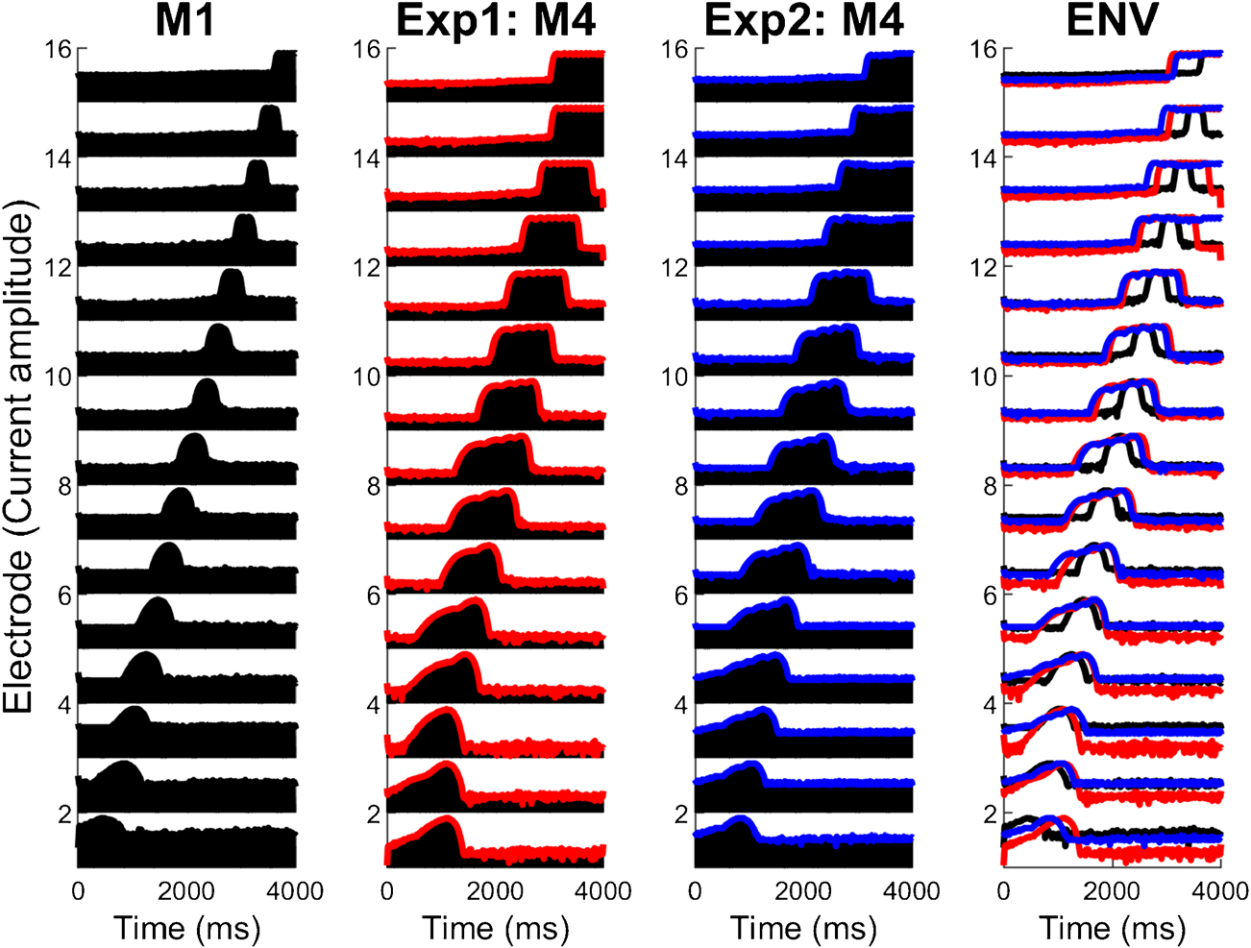
Comparison of blurring at the input (exp. 1) and at the output (exp. 2). Electrodograms for a chirp sound for M1, M4 from exp. 1 (envelope in red), M4 from exp. 2 (envelope in blue) and all three envelopes overlaid

Blurring by a factor of up to 3 did not increase SRTs, either when applied at the input (experiment 1) or electrode (experiment 2) stage, but there were highly significant effects at group level for blurring factors of 4 and higher. As shown in Fig. 8, the relationship between SRT and the amount of blurring was very similar for the two experiments at group level when blurring all fifteen channels. It is worth noting that although both manipulations were applied in the spectral/place-of excitation domain, they both also have the effect of blurring the temporal representation of the stimulus. This is illustrated in Fig. 9, which shows electrodograms in response to a chirp, for M1 and with a four-fold blurring (M4) as applied in experiments 1 and 2. Indeed, the electrodograms look quite similar for the manipulations in the two experiments, although it should be noted that in experiment 1 stimulation was always interleaved across electrodes, whereas in experiment 2 it was presented simultaneously on subsets of adjacent electrodes. This difference is not visible in Fig. 8 due to the coarse time-scale used.

The pattern of results shown in Fig. 8, where SRTs are constant up to a blurring factor of about 3, is consistent with previous studies that evaluated double-electrode stimulation in CI users and reported similar outcomes to single-electrode stimulation (Hughes et al. 2013; Goehring et al. 2014). It is also consistent with the idea that smaller amounts of blurring have no effect because they are smaller than any spread of excitation or degradation of the input signal that occurs in the listener’s CI with a standard clinical setting. This “internal” blurring could result from the current spread produced by individual electrodes, and/or degradations in the spectral representation at more central stages of the auditory system. As noted in the Introduction, this general idea, whereby sensitivity is measured as a function of the amount of external distortion, has been used in the equivalent noise method to shed light on perceptual limitations in previous research (Lu and Dosher 1998; Pelli and Farell 1999; Ihlefeld and Sanes 2015). Although the equivalent noise method differs from the approach used here in some important respects—for example by using signal-detection theory to model simple detection and discrimination tasks—both approaches have the potential to tease apart the effects of peripheral noise and more central processing efficiency. In terms of the broken-stick fits to our individual data from experiment 1 (Figs. 3 and 4a), we interpret the knee point as a measure of the listener’s intrinsic spectro-temporal limitations, and assume that the SRT for any given map is affected both by the spectro-temporal contrast and by more central factors, including the ability to use linguistic knowledge and context to disambiguate a degraded signal. In support of the second hypothesis, the knee point correlated with performance on the STRIPES measure of spectro-temporal processing. An un-tested prediction is that applying a cognitive manipulation, such as requiring the listener to perform a concurrent competing task, would increase the SRT overall but not alter the knee point.

### Blurring Applied to 5 Out of 15 Electrodes

There was no effect of blurring on speech-in-noise perception when one-third of the active electrode channels were manipulated. This was an unexpected finding, especially for the most extreme conditions that used 8-fold wider analysis filters or 8 simultaneously stimulated electrodes in the 5-of-15 condition. Furthermore, experiment 2 provided no evidence that deselecting the to-be-blurred channels had an effect on speech-in-noise perception. This may lead one to the conclusion that moderate numbers of highly degraded channels do not degrade speech perception, and that there is nothing to be gained by turning them off. This conclusion should be qualified by considering the differences between our simulations of bad channels and those that occur in everyday CI use, and by the particular testing materials that we used.

The manipulations that we performed were limited to the effects of spectral blurring and some small shifts of the centre frequencies in the experimental MAPs, but did not include distortions such as those caused by cross-turn stimulation (Frijns et al. 1995; Cosentino et al. 2015a), ectopic stimulation (Finley et al. 1990), and neural dead regions (Bierer and Faulkner 2010; Cosentino et al. 2015b). These distortions manifest as place-pitch reversals (Kenway et al. 2015) or double-peaked excitation patterns, rather than the simple reduction in spectral contrast that is produced by blurring. Speech perception may also be impacted by poor temporal representation of information, as evidenced, for example, by across-electrode differences in modulation detection thresholds, rate discrimination, or gap detection (Garadat et al. 2013; Bierer et al. 2015; Cosentino et al. 2016). We do not know how robust speech perception is to these types of distortions, occurring either on all or on a subset of electrodes.

The blurring used here was applied to evenly spaced electrodes. The information conveyed by adjacent electrodes will be highly correlated, and so if (as appears to be the case) listeners can ignore degraded information on some electrodes (as in experiment 1), a more accurate representation of that information may be provided by nearby electrodes. It is likely that, if we had blurred five adjacent electrodes, particularly those driven by frequency regions that are particularly important for speech, some deficits would have been observed. We chose to space the blurred channels evenly, because most channel deselection methods impose some degree of spacing between deselected channels. It is possible that those methods would be more effective, at least after a period of acclimatization, if deactivation of adjacent electrodes were less constrained.

Finally, the speech test we used employed a masking noise based on a competing male talker. As this is one of the most difficult masker conditions for CI users, it resulted in highly positive overall SNRs for the SRTs in the SIN test for all subjects. These SRTs are higher than observed in studies with more- stationary and less-sparse maskers (e.g. Goehring et al. 2017, 2019a). Therefore, the high SNR conditions for the SIN test may have led to reduced effects of blurring than if low SNR conditions had been tested. However, the competing talker noise is characterized by sparse but highly energetic spectral components typical for speech that can strongly interfere with the target speech and this type of noise has been reported to be more sensitive to differences in spectral resolution than other types in a recent study (Croghan and Smith 2018). The reason for this can be understood by considering an input signal consisting of a tone masked by a flat-spectrum noise. Doubling the width of the analysis filter would change the signal-to-noise ratio at the output by 3 dB, but if the noise were spectrally remote from the tone then a much larger difference would occur. Nevertheless, we plan to investigate if the (lack of) effect of blurring also holds for other types of maskers that lead to lower SNR conditions for the SRTs.

Despite the above limitations and caveats, we believe that the results found with blurring have important implications for optimization strategies for CIs with relevance to channel interaction, site-selection and speech-in-noise perception. Specifically, CI listeners’ speech perception in a competing talker noise was unchanged even with extreme amounts of spectral blurring on, or the deactivation of, a moderate number of evenly spaced electrodes. This finding should be taken into account when designing strategies that aim to optimize channel interaction by manipulating a subset of electrodes that are evenly spaced along the array. Our results also suggest that deselection strategies may need to deactivate sets of adjacent electrodes, contrary to most approaches adopted so far, at least when the basis for deselection is expected to be a wider-than-average excitation pattern. Finally, the development of channel deselection strategies should consider the need to identify distortions different from those modelled here, and/or to disable more or more-closely-spaced channels.

For the goal of reducing channel interaction in CIs to achieve better outcomes, studies using blurring or similar controlled manipulations of channel interaction can lead to a better understanding of the associated effects on speech perception and inform future interventions. For example, spectral blurring or similar approaches could be used as a tool to assess individual effects of channel interaction relevant to speech perception by measuring blurring knee points as an estimate of the spectro-temporal resolution of individual listeners, independently of any cognitive effects on the overall level of performance. It could also, for example, demonstrate whether novel modes of stimulation or coding strategies improve spectro-temporal resolution.

## Conclusion

Channel interaction was manipulated in CI users with two types of spectral blurring, at the level of the analysis filters and at the level of the electrodes of the CI. We found a main effect of spectral blurring on SIN performance in CI subjects for the case when all electrodes were blurred. The degree to which blurring affected each subject’s individual SIN performance was associated with their performance on a spectro-temporal test and their SIN performance with the map similar to their everyday map. These associations were in the predicted direction with better-performing subjects being affected by smaller amounts of blurring. Surprisingly, when 5 evenly spaced electrodes were manipulated there was no effect even for extreme amounts of blurring. The findings were consistent between the two types of blurring for which experiments were performed several months apart. Importantly, there was again no effect on performance when the five blurred electrode channels were deactivated in the second experiment. The results show that speech-in-noise performance by experienced CI users was unchanged by spectro-temporal distortions imposed by blurring or by deactivating a subset of electrodes evenly spaced along the array, and have implications for the design and implementation of channel deselection strategies.

## References

[CR1] Anderson ES, Nelson DA, Kreft H, Nelson PB, Oxenham AJ (2011). Comparing spatial tuning curves, spectral ripple resolution, and speech perception in cochlear implant users. J Acoust Soc Am.

[CR2] Archer-Boyd AW, Southwell R, Deeks JM, Turner RE, Carlyon RP (2018). Development and validation of a spectro-temporal processing test for cochlear-implant listeners. J Acoust Soc Am.

[CR3] Archer-Boyd A, Goehring T, Carlyon RP (2020) The effect of free-field presentation and processing strategy on a measure of spectro-temporal processing by cochlear-implant listeners. PsyArXiv https://psyarxiv.com/m4drj/10.1177/2331216520964281 PMC7734493 33305696

[CR4] Bench J, Kowal Å, Bamford J (1979). The BKB (Bamford-Kowal-Bench) sentence lists for partially-hearing children. Br J Audiol.

[CR5] Berenstein CK, Mens LHM, Mulder JJS, Vanpoucke FJ (2008). Current steering and current focusing in cochlear implants: comparison of monopolar, tripolar, and virtual channel electrode configurations. Ear Hear.

[CR6] Berg KA, Noble JH, Dawant BM, Dwyer RT, Labadie RF, Gifford RH (2019). Speech recognition as a function of the number of channels in perimodiolar electrode recipients. J Acoust Soc Am.

[CR7] Berg KA, Noble JH, Dawant B, Dwyer R, Labadie R, Gifford RH (2019). Effect of number of channels and speech coding strategy on speech recognition in mid-scala electrode recipients. J Acoust Soc Am.

[CR8] Bierer JA (2007). Threshold and channel interaction in cochlear implant users: evaluation of the tripolar electrode configuration. J Acoust Soc Am.

[CR9] Bierer JA, Faulkner KF (2010). Identifying cochlear implant channels with poor electrode-neuron interface: partial tripolar, single-channel thresholds and psychophysical tuning curves. Ear Hear.

[CR10] Bierer JA, Litvak L (2016). Reducing channel interaction through cochlear implant programming may improve speech perception: current focusing and channel deactivation. Trends Hear.

[CR11] Bierer JA, Deeks JM, Billig AJ, Carlyon RP (2015) Comparison of signal and gap-detection thresholds for focused and broad cochlear implant electrode configurations. J Assoc Res Otolaryngol 16(2):273–284 10.1007/s10162-015-0507-y PMC4368655 25644786

[CR12] Bingabr M, Espinoza-Varas B, Loizou PC (2008). Simulating the effect of spread of excitation in cochlear implants. Hear Res.

[CR13] Bonham BH, Litvak LM (2008). Current focusing and steering: modeling, physiology, and psychophysics. Hear Res.

[CR14] Brochier T, Guerit F, Garcia C, Deeks JM, Bance ML, Carlyon RP (2020) Evaluating and comparing behavioural and electrophysiological estimates of neural health in cochlear implant users. PsyArXiv psyarxiv.com/2kp7x 10.1007/s10162-020-00773-0 PMC7822986 33150541

[CR15] Carlyon RP, Long CJ, Deeks JM, McKay CM (2007). Concurrent sound segregation in electric and acoustic hearing. J Assoc Res Otolaryngol.

[CR16] Cosentino S, Gaudrain E, Deeks JM, Carlyon RP (2015). Multistage nonlinear optimization to recover neural activation patterns from evoked compound action potentials of cochlear implant users. IEEE Trans Biomed Eng.

[CR17] Cosentino S, Deeks JM, Carlyon RP (2015). Procedural factors that affect psychophysical measures of spatial selectivity in cochlear implant users. Trends Hear.

[CR18] Cosentino S, Carlyon RP, Deeks JM, Parkinson W, Bierer JA (2016). Rate discrimination, gap detection and ranking of temporal pitch in cochlear implant users. J Assoc Res Otolaryngol.

[CR19] Crew JD, Galvin JJ (2012). Channel interaction limits melodic pitch perception in simulated cochlear implants. J Acoust Soc Am.

[CR20] Croghan NBH, Smith ZM (2018). Speech understanding with various maskers in cochlear-implant and simulated cochlear-implant hearing: effects of spectral resolution and implications for masking release. Trends iHear.

[CR21] Croghan NBH, Duran SI, Smith ZM (2017). Re-examining the relationship between number of cochlear implant channels and maximal speech intelligibility. J Acoust Soc Am.

[CR22] Cullington HE, Zeng F-G (2008). Speech recognition with varying numbers and types of competing talkers by normal-hearing, cochlear-implant, and implant simulation subjects. J Acoust Soc Am.

[CR23] Dawson PW, Mauger SJ, Hersbach AA (2011). Clinical evaluation of signal-to-noise ratio--based noise reduction in nucleus®cochlear implant recipients. Ear Hear.

[CR24] De Jong MAM, Briaire JJ, Frijns JHM (2019). Dynamic current focusing: a novel approach to loudness coding in cochlear implants. Ear Hear.

[CR25] Deeks JM, Carlyon RP (2004). Simulations of cochlear implant hearing using filtered harmonic complexes: implications for concurrent sound segregation. J Acoust Soc Am.

[CR26] DeVries L, Arenberg JG (2018). Current focusing to reduce channel interaction for distant electrodes in cochlear implant programs. Trends Hear.

[CR27] Dorman MF, Loizou PC (1997). Speech intelligibility as a function of the number of channels of stimulation for normal-hearing listeners and patients with cochlear implants. Am J Otol.

[CR28] Finley CC, Wilson BS, White MW (1990) Models of neural responsiveness to electrical stimulation. In: Cochlear implants. Springer, pp 55–96

[CR29] Friesen LM, Shannon RV, Baskent D, Wang X (2001). Speech recognition in noise as a function of the number of spectral channels: comparison of acoustic hearing and cochlear implants. J Acoust Soc Am.

[CR30] Frijns JHM, De Snoo SL, Schoonhoven R (1995). Potential distributions and neural excitation patterns in a rotationally symmetric model of the electrically stimulated cochlea. Hear Res.

[CR31] Fu Q-J, Nogaki G (2005). Noise susceptibility of cochlear implant users: the role of spectral resolution and smearing. J Assoc Res Otolaryngol.

[CR32] Fu Q-J, Shannon RV (2002). Frequency mapping in cochlear implants. Ear Hear.

[CR33] Garadat SN, Zwolan TA, Pfingst BE (2012) Across-site patterns of modulation detection: Relation to speech recognition. J Acoust Soc Am 131(5):4030–4041 10.1121/1.3701879 PMC3356319 22559376

[CR34] Garadat SN, Zwolan TA, Pfingst BE (2013). Using temporal modulation sensitivity to select stimulation sites for processor MAPs in cochlear implant listeners. Audiol Neurotol.

[CR35] Goehring JL, Neff DL, Baudhuin JL, Hughes ML (2014). Pitch ranking, electrode discrimination, and physiological spread-of-excitation using cochlear’s dual-electrode mode. J Acoust Soc Am.

[CR36] Goehring T, Bolner F, Monaghan JJM, van Dijk B, Zarowski A, Bleeck S (2017). Speech enhancement based on neural networks improves speech intelligibility in noise for cochlear implant users. Hear Res.

[CR37] Goehring T, Keshavarzi M, Carlyon RP, Moore BCJ (2019). Using recurrent neural networks to improve the perception of speech in non-stationary noise by people with cochlear implants. J Acoust Soc Am.

[CR38] Goehring T, Archer-Boyd A, Deeks JM, Arenberg JG, Carlyon RP (2019). A site-selection strategy based on polarity sensitivity for cochlear implants: effects on spectro-temporal resolution and speech perception. J Assoc Res Otolaryngol.

[CR39] Grange JA, Culling JF, Harris NSL, Bergfeld S (2017). Cochlear implant simulator with independent representation of the full spiral ganglion. J Acoust Soc Am.

[CR40] Hanekom JJ, Shannon RV (1998). Gap detection as a measure of electrode interaction in cochlear implants. J Acoust Soc Am.

[CR41] Henry BA, McKay CM, McDermott HJ, Clark GM (2000). The relationship between speech perception and electrode discrimination in cochlear implantees. J Acoust Soc Am.

[CR42] Hersbach AA, Arora K, Mauger SJ, Dawson PW (2012). Combining directional microphone and single-channel noise reduction algorithms: a clinical evaluation in difficult listening conditions with cochlear implant users. Ear Hear.

[CR43] Hughes ML, Stille LJ, Baudhuin JL, Goehring JL (2013). ECAP spread of excitation with virtual channels and physical electrodes. Hear Res.

[CR44] Ihlefeld S, Sanes D (2015) Increased internal noise following juvenile hearing loss. At Midwinter Meeting Association for Research in Otolaryngology, 38, 277, Baltimore, US

[CR45] Jahn KN, DiNino M, Arenberg JG (2019). Reducing simulated channel interaction reveals differences in phoneme identification between children and adults with normal hearing. Ear Hear.

[CR46] Kenway B, Tam YC, Vanat Z, Harris F, Gray R, Birchall J, Carlyon R, Axon P (2015). Pitch discrimination: an independent factor in cochlear implant performance outcomes. Otol Neurotol.

[CR47] Langner F, Saoji AA, Büchner A, Nogueira W (2017). Adding simultaneous stimulating channels to reduce power consumption in cochlear implants. Hear Res.

[CR48] Litvak LM, Spahr AJ, Saoji AA, Fridman GY (2007). Relationship between perception of spectral ripple and speech recognition in cochlear implant and vocoder listeners. J Acoust Soc Am.

[CR49] Loizou PC, Poroy O, Dorman M (2000). The effect of parametric variations of cochlear implant processors on speech understanding. J Acoust Soc Am.

[CR50] Lu Z-L, Dosher BA (1998). External noise distinguishes attention mechanisms. Vis Res.

[CR51] MacLeod A, Summerfield Q (1990). A procedure for measuring auditory and audiovisual speech-reception thresholds for sentences in noise: rationale, evaluation, and recommendations for use. Br J Audiol.

[CR52] Mens LHM, Berenstein CK (2005). Speech perception with mono-and quadrupolar electrode configurations: a crossover study. Otol Neurotol.

[CR53] Mesnildrey Q, Macherey O (2015). Simulating the dual-peak excitation pattern produced by bipolar stimulation of a cochlear implant: effects on speech intelligibility. Hear Res.

[CR54] Nelson DA, Kreft HA, Anderson ES, Donaldson, GS (2011) Spatial tuning curves from apical, middle, and basal electrodes in cochlear implant users. J Acoust Soc Am 129(6):3916–3933 10.1121/1.3583503 PMC3135148 21682414

[CR55] Noble JH, Labadie RF, Gifford RH, Dawant BM (2013). Image-guidance enables new methods for customizing cochlear implant stimulation strategies. IEEE Trans Neural Syst Rehabil Eng.

[CR56] Noble JH, Gifford RH, Hedley-Williams AJ, Dawant BM, Labadie RF (2014). Clinical evaluation of an image-guided cochlear implant programming strategy. Audiol Neurotol.

[CR57] Oxenham AJ, Kreft HA (2014). Speech perception in tones and noise via cochlear implants reveals influence of spectral resolution on temporal processing. Trends Hear.

[CR58] Pelli DG, Farell B (1999). Why use noise?. JOSA A.

[CR59] Qin MK, Oxenham AJ (2003). Effects of simulated cochlear-implant processing on speech reception in fluctuating maskers. J Acoust Soc Am.

[CR60] Rothauser EH (1969). IEEE recommended practice for speech quality measurements. IEEE Trans Audio Electroacoust.

[CR61] Saleh SM, Saeed SR, Meerton L, Moore DR, Vickers DA (2013). Clinical use of electrode differentiation to enhance programming of cochlear implants. Cochlear Implants Int.

[CR62] Schvartz-Leyzac KC, Zwolan TA, Pfingst BE (2017). Effects of electrode deactivation on speech recognition in multichannel cochlear implant recipients. Cochlear Implants Int.

[CR63] Srinivasan, A. G., Padilla, M., Shannon, R. V, & Landsberger, D. M. (2013). Improving speech perception in noise with current focusing in cochlear implant users. Hear Res, 299, 29–36 10.1016/j.heares.2013.02.004 PMC3639477 23467170

[CR64] van den Honert C, Kelsall DC (2007). Focused intracochlear electric stimulation with phased array channels. J Acoust Soc Am.

[CR65] Vickers D, Degun A, Canas A, Stainsby T, Vanpoucke F (2016) Deactivating cochlear implant electrodes based on pitch information for users of the ACE strategy. In: Physiology, psychoacoustics and cognition in normal and impaired hearing. Springer, p 115–123 10.1007/978-3-319-25474-6_13 27080652

[CR66] Zhou N (2016) Monopolar detection thresholds predict spatial selectivity of neural excitation in cochlear implants: Implications for speech recognition. PLoS One, 11(10) 10.1371/journal.pone.0165476 PMC5087957 27798658

[CR67] Zhou N (2017). Deactivating stimulation sites based on low-rate thresholds improves spectral ripple and speech reception thresholds in cochlear implant users. J Acoust Soc Am.

[CR68] Zwolan TA, Collins LM, Wakefield GH (1997). Electrode discrimination and speech recognition in postlingually deafened adult cochlear implant subjects. J Acoust Soc Am.

